# A Simple Model to Assess the Probability of Invasion in Ductal Carcinoma In Situ of the Breast Diagnosed by Needle Biopsy

**DOI:** 10.1155/2014/480840

**Published:** 2014-07-08

**Authors:** Oldřich Coufal, Iveta Selingerová, Pavlína Vrtělová, Petr Krsička, Lucie Gabrielová, Pavel Fabian, Kateřina Stískalová, Monika Schneiderová, Alexandr Poprach, Ivan Justan

**Affiliations:** ^1^Department of Surgical Oncology, Masaryk Memorial Cancer Institute, Zluty kopec 7, 656 53 Brno, Czech Republic; ^2^Department of Comprehensive Cancer Care, Masaryk Memorial Cancer Institute, Zluty kopec 7, 656 53 Brno, Czech Republic; ^3^Department of Mathematics and Statistics of the Faculty of Science, Masaryk University, Kotlarska 267/2, 611 37 Brno, Czech Republic; ^4^Department of Pathology, Masaryk Memorial Cancer Institute, Zluty kopec 7, 656 53 Brno, Czech Republic; ^5^Department of Radiology, Masaryk Memorial Cancer Institute, Zluty kopec 7, 656 53 Brno, Czech Republic

## Abstract

*Objectives*. The aim of the study was to develop a clinical prediction model for assessing the probability of having invasive cancer in the definitive surgical resection specimen in patients with biopsy diagnosis of ductal carcinoma in situ (DCIS) of the breast, to facilitate decision making regarding axillary surgery. *Methods*. In 349 women with DCIS, predictors of invasion in the definitive resection specimen were identified. A model to predict the probability of invasion was developed and subsequently simplified to divide patients into two risk categories. The model's performance was validated on another patient population. *Results*. Multivariate logistic regression revealed four independent predictors of invasion: (i) suspicious (micro)invasion in the biopsy specimen; (ii) visibility of the lesion on ultrasonography; (iii) size of the lesion on mammography >30 mm; (iv) clinical palpability of the lesion. The actual frequency of invasion in the high-risk patient group in the test and validation population was 52.6% and 48.3%, respectively; in the low-risk group it was 16.8% and 7.1%, respectively. *Conclusion*. The model proved to have good performance. In patients with a low probability of invasion, an axillary procedure can be omitted without a substantial risk of additional surgery.

## 1. Introduction

In patients with biopsy diagnosis of ductal carcinoma in situ (DCIS) of the breast, an invasive cancer is often found in the definitive surgical resection specimen. The frequency of such upstaging varies in different sets of patients and is usually between 15% and 40%, with a median value of 26.0% [1]. Though the incidence of nodal metastases in pure DCIS is rare (3.7% in a meta-analysis [2]), the role of sentinel lymph node biopsy (SLNB) has been debated, because of the possibility of invasion in the definitive surgical specimen, and there are substantial differences regarding its indication worldwide [3, 4]. SLNB is not considered a demanding procedure, but there are still some associated costs and inconvenience for the patient. The frequency of lymphoedema occurring three years after SLNB can be as high as 8% [5]. Thus, routine SLNB in all patients with a biopsy diagnosis of DCIS seems inappropriate. With additional SLNB performed selectively only in cases with subsequent detection of invasion, about one-third of women would be exposed to further surgery. It would be of clinical benefit therefore to stratify patients with a biopsy diagnosis of DCIS before surgery, according to the probability of later upstaging. SLNB could be reserved only for patients with a high probability of having invasive cancer and could otherwise be omitted.

Several authors have attempted to identify variables that could serve as predictors of invasive carcinoma in the resection specimen of patients with DCIS diagnosed by biopsy. Although some have failed to identify any reliable predictor [6], in the majority of the studies variables of predictive value have been found; these refer to different clinicopathologic items such as the age of the patient [7], the palpability of the lesion [8–12], the characteristics of the lesion on imaging methods (mass/microcalcifications/others) [8–10, 12–15], the size of the lesion [7, 10, 11, 14], the type of biopsy (core cut/vacuum assisted/excisional) [7, 8, 16], and histopathologic findings in the biopsy specimen (proportion of affected ducts, grade, suspicious invasion) [7, 8, 10, 12]. A more detailed review of the literature on this topic has been published by Brennan et al. [1]. The findings are not fully consistent across the studies and sometimes variables were used that may not be routinely available in the clinical setting (e.g., the size of the lesion on magnetic resonance imaging [11], or that may not be reproducible, owing to local differences in the diagnostic algorithm (e.g., type of biopsy [7, 8, 16]).

The aim of this study was to develop a clinical prediction model for assessing the probability of having invasive cancer in surgical resection specimens in patients diagnosed with DCIS by needle biopsy. The model should be simple to use and include only variables that are generally available in the clinical setting and that are preferably not subject to local differences in the diagnostic procedure.

## 2. Methods

### 2.1. Test Population and Construction of the Predictive Model

Inclusion criteria for the test population of the study were as follows: (1) DCIS of the breast diagnosed by needle biopsy and (2) resection of the lesion by partial or total mastectomy in the Masaryk Memorial Cancer Institute, Brno, Czech Republic (MOU) between 2006 and 2011. There were no specific exclusion criteria. The participants were identified retrospectively from the database of the MOU.

For the 349 consecutive patients fulfilling the inclusion criteria, clinicopathologic variables available before surgery that, according to published data and the authors' expectations, may be related to the probability of invasive cancer being detected in the definitive surgical specimen were recorded. The variables were collected retrospectively from usual medical records and they are listed with their categories in Table 1.

- The age was calculated as a difference between the year of the surgery and the year of the birth date of the patient.
- Assessment of the palpability of the lesion (not palpable/palpable) was at the discretion of the physician performing the physical examination.
- Size of the lesion on mammography was measured by the radiologist as the maximum diameter of the lesion on the standard mediolateral oblique (MLO) or craniocaudal (CC) mammographic views. In multifocal lesions, the diameter of the whole pathologic area was used for calculations, not the size of the largest focus.
- Assessment of the characteristics of the lesion on mammography (presence of microcalcifications and/or a mass or distortion on the mammogram) and visibility of the lesion on ultrasonography were at the discretion of the examining radiologist.
- The biopsy device used and the type of image guidance were chosen by the interventional radiologists performing the biopsy. Usually, but not always, lesions visible on ultrasonography were biopsied using the 14-Gauge automated device under ultrasonographic guidance.
- Histologic grade in the biopsy specimen was assessed by the pathologist as the nuclear grade according to the Nottingham grading system for invasive cancer. Lesions with G1 and G2 nuclei were considered nonhigh-grade and lesions with G3 nuclei were considered high-grade. Generally, the high grade DCIS exhibit nuclei with diameters >2.5 RBC equivalents, pleomorphism with irregular nuclear contour, coarse clumped and vesicular chromatin, one or more prominent nucleoli, and frequent mitotic figures. Presence or absence of necrosis was not taken into account.
- Suspect (micro)invasion in the biopsy specimen was assessed by the pathologist. It was defined as the presence of suspicious trabecular tumor cell formation in the context of stromal response (e.g., myofibroblastic proliferation and/or dense lymphoid infiltration), which cannot be unequivocally classified as either invasive carcinoma or lobular extension of DCIS, even if the immunohistochemical detection of myoepithelial markers has been performed.

The basic characteristics of patients and their parameters were summarized using frequency tables and descriptive statistics. Association of the considered variables with subsequent detection of invasion in the resection specimen (predictive value) was assessed by univariate analysis, using the Pearson chi-square test and the Fisher exact test for categorical data. Factors showing predictive value in univariate analysis were evaluated by multivariate analysis in cases where all of these factors were available. The cut-off *P* value used for selecting variables from univariate analysis to multivariate analysis was 0.1. The sample size was sufficient for regression modeling as we were interested in six potential predictors with more than ten-fold number of outcome events in the analyzed subpopulation. A binary logistic regression with backward elimination method was used to construct a model to assess the probability of having invasive cancer in the definitive resection specimen. The significance level used for a variable to stay in the model was 0.05. To make the model more user-friendly the score number was assigned to each variable as the corresponding Beta coefficient from the multivariate regression model multiplied by 10. The performance of the model was assessed by concordance statistics and area under ROC curve. The model was subsequently simplified to enable easy sorting of patients into two risk groups, according to the probability of invasion in the definitive specimen. The probability of invasion according to the simplified predictive model was assessed for every patient in the test population and compared to the real histopathologic finding in the resection specimen.

### 2.2. Validation of the Model

Subsequently, consecutive patients with DCIS diagnosed by needle biopsy, who underwent resection of the primary lesion at MOU between January 2012 and March 2013 (validation population), were identified. In these patients, the clinicopathologic variables used in the predictive model developed were retrospectively collected from usual medical records. Homogeneity in the test and validation populations was validated using Pearson's chi-square test. The performance of the predictive model was assessed by concordance statistics and area under ROC curve. The probability of invasion according to the simplified model was assessed for every patient of the validation population and compared to the real histopathologic finding in the resection specimen.

Statistical analyses were performed with Statistica (Version 10) and SAS Enterprise Guide (Version 5.1).

## 3. Results

### 3.1. Test Population and Construction of the Predictive Model

The test population was 349 patients. Their median age was 57 years (range: 22–86 years) and invasion (or microinvasion) in the resection specimen was detected in 118 cases (33.8%). The results of univariate analysis assessing the relationship between particular clinicopathologic variables and the detection of invasion are shown in Table 1.

Multivariate logistic regression analysis on 204 patients revealed four independent predictors of invasive cancer on final pathology: (i) suspicious invasion (or microinvasion) in the biopsy specimen; (ii) visibility of the lesion on ultrasonography; (iii) size of the lesion on mammography >30 mm; (iv) clinical palpability of the lesion.

Using these results, a model to predict the probability of having invasive cancer was developed. The appropriate equation for calculating the probability is

(1) p=1 ×(1+exp⁡(2.37−1.33∗suspI−1.06∗usg− 0.92∗size−0.92∗palpable))−1,

where

- susp*I* = 1 if there is present suspect (micro)invasion in the biopsy specimen (otherwise susp*I* = 0);
- usg = 1 if the lesion is visible on ultrasonography (otherwise usg = 0);
- size = 1 if the size of the lesion on mammography is >30 mm (otherwise size = 0);
- palpable = 1 if the lesion is palpable (otherwise palpable = 0).

According to the user-friendly version of the model, the probability of invasion in an individual case is a function of total score, which is calculated as a sum of specific score numbers for corresponding variables. The specific score numbers for each variable used are listed in Table 2.

The probability of invasion related to the total score is shown in Table 3.

The performance of the predictive model in the test population is shown in Table 4.

### 3.2. Simplification of the Model

A probability of invasion equal to 0.25 was set as a cut-off between the “low-risk” and the “high-risk” groups of patients. This cut-off was chosen as clinically relevant with the priority to increase the negative predictive value of the model. The corresponding score number equals 12.6 (see Table 3). The presence of suspicious (micro)invasion in the biopsy specimen has a specific score number of 13.3 (see Table 2), which by itself predicts a probability of invasion greater than 0.25. Therefore, suspicious (micro)invasion is considered a “major risk factor.” The specific score numbers for other variables used in the model are less than 12.6 (see Table 2); they were termed “minor risk factors.” The presence of only one of these factors gives a probability of invasion under 0.25. In order to divide the whole population into two risk groups, a simple rule has been set:

- if there is a maximum of one minor risk factor present, then the probability of invasion in the resection specimen is low (under 0.25);
- if there is suspicious (micro)invasion in the biopsy specimen, or two or three minor risk factors are present, then the probability of invasion in the resection specimen is high (above 0.25).

The rule is highlighted in Box 1.

The performance of the simplified model in the test population is shown in Table 5.

### 3.3. Validation of the Simplified Model

The validation population consisted of 71 patients. Their median age was 60 years (range: 32–79 years) and invasion (or microinvasion) in the resection specimen was detected in 17 cases (23.9%). Homogeneity in the test and validation population was not rejected at a 0.05 level of significance, suggesting that both populations were comparable in terms of the predictive factors and invasion in the resection specimen. The performance of the predictive model in the validation population is shown in Table 6.

The performance of the simplified model in the validation population is shown in Table 7.

## 4. Discussion

In this study, medical records from 420 patients with DCIS diagnosed by needle biopsy were analysed. Although there were some missing data, still it is one of the largest monoinstitutional studies on this topic to date. It confirmed the predictive value of some clinicopathologic variables in terms of upstaging to invasive cancer. Furthermore, this study has added to the body of research by developing a comprehensive but simple model, with clear-cut results assigning patients into two categories according to their probability of having invasive cancer in the resection specimen.

Contrary to expectations, the predictive value of some variables was not confirmed in the analysed patient population. Most notably, there was no association between invasion in the resection specimen and mammographic appearance of the lesion (mass or distortion and/or microcalcifications). This may have been caused by inappropriate description of the mammographic findings in the usual medical records. However, available mammographic images (from the majority of patients) were reviewed by a single radiologist, with no significant changes in the results. Further, no association was found between upstaging and the thickness of the needle used to obtain the biopsy. The possible association found in other series [7, 8, 16] may be related to the amount of tissue sampled and histologically examined. In this respect, the number of samples would also be of importance but this variable was not analysed in the present study. The choice of needle may ultimately be influenced by the preferences of the performing radiologist and hence may be biased.

When constructing the model, the variables to be included were carefully considered. Even if the most comprehensive model with six possible variables was built up, the area under the ROC curve for the test population did not go above 0.8 and it was substantially lower in the validation population. Thus, simplicity was preferred, and one variable was used from each basic diagnostic component: histology of the biopsy specimen (suspected invasion); ultrasonography (visibility); mammography (size); clinical examination (palpability). Different values for the cut-off point used to compare smaller with larger lesions were tested; finally, a value of 30 mm for the cut-off appeared to be the best with regard to the performance of the model and its clinical use.

Park et al. recently published their nomogram for predicting underestimation of invasiveness in DCIS diagnosed by preoperative needle biopsy [17]. The results are similar to the present ones in some aspects. Their model includes suspicious (micro)invasion in the biopsy specimen, palpability of the lesion, characteristics of the lesion on ultrasonography, and the method of biopsy. The ROC of their model reached 0.75. However, their nomogram was not suitable for the population used in the present study, as, in most cases, the detailed characteristics of the lesion on ultrasonography (mass and/or calcifications) were not clear from the usual medical records of the study population. Contrary to the findings of Park et al., in the present study population, the method of biopsy showed no predictive value, as discussed above.

There are some limitations to this study. In some patients, the appropriate data were lacking. Not every woman had both basic imaging methods (mammography and ultrasonography) performed before undergoing surgery. Sometimes the ultrasonography was performed after the stereotactic biopsy, so the resulting haematoma made it impossible to decide whether the lesion by itself would have been visible or not. In some patients the exact size of the lesion in millimetres was not recorded in the medical notes. The diameter was measured by reviewing the mammographic images but, in some patients diagnosed outside MOU, these were not available. Sometimes the information about grade was lacking in the histopathologic report of the biopsy and it was not possible to review the biopsy slides from patients with the original histopathologic examination performed outside our institution. Finally, for some cases, even though all the data were completely recorded, it was impossible to decide how some variables should be categorized (e.g., one type of examination performed by two different clinicians with discordant results). But, on the other hand, the real clinical conditions led to construction of a model that is relevant to actual clinical practice and would be applicable to the majority of patients.

## 5. Conclusions

In conclusion, through one of the largest monoinstitutional studies on this topic, a model has been developed to predict the probability of invasive cancer in patients with needle biopsy of DCIS. The model demonstrated good performance in both test and validation population. It is quite simple and gives clear-cut result in terms of low or high probability of invasion. The cut-off value has been selected so that, for patients with a low probability of invasion, an axillary procedure can be omitted without substantial risk of their needing to undergo additional surgery, while patients with a high probability of invasion can be offered up-front SLNB. As the variables used in the model were selected with respect to their clinical use, it should be universally applicable. Validation of the model in independent patient populations is desirable.

## Figures and Tables

**Box 1:**
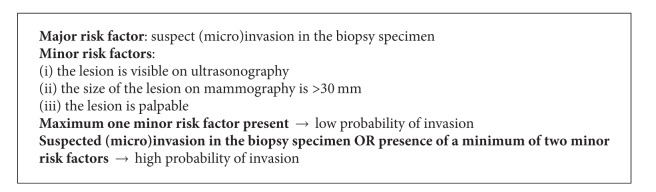
A simplified model to predict the probability of invasion in ductal carcinoma in situ of the breast diagnosed by needle biopsy.

**Table 1:** Clinicopathologic variables and their association with the incidence of invasion detected in the surgical specimen on univariate analysis.

Variable	Category	Number of patients with pure DCIS (%), *n* = 231	Number of patients with detected invasion in the resection specimen (%), *n* = 118	Odds ratio (95% CI)	Significance (*P* value)
Age, *n* = 349	>60 years ≤60 years	83 (61.9) 148 (68.8)	51 (38.1) 67 (31.2)	1.357 (0.863, 2.134)	0.202

Palpability of the lesion, *n* = 348	Palpable Not palpable	53 (49.5) 177 (73.4)	54 (50.5) 64 (26.6)	2.818 (1.753, 4.530)	<0.001

Characteristics of the lesion on mammography, *n* = 322	Mass or distortion Microcalcifications (only)	50 (66.7) 169 (68.4)	25 (33.3) 78 (31.6)	1.083 (0.625, 1.878)	0.779

Size of the lesion on mammography, *n* = 290	>30 mm ≤30 mm	55 (55.6) 142 (74.3)	44 (44.4) 49 (25.7)	2.318 (1.389, 3.870)	0.001

Visibility of the lesion (mammographically detected) on ultrasonography, *n* = 250	Visible Not visible	116 (58.6) 44 (84.6)	82 (41.4) 8 (15.4)	3.888 (1.739, 8.693)	<0.001

Biopsy device, *n* = 319	14-Gauge automated 11-Gauge vacuum assisted biopsy	177 (66.0) 37 (72.5)	91 (34.0) 14 (27.5)	1.359 (0.699, 2.642)	0.419

Image guidance, *n* = 319	Ultrasonographic Stereotactic	64 (56.1) 150 (73.2)	50 (43.9) 55 (26.8)	2.131 (1.316, 3.450)	0.003

Histologic grade (in the biopsy specimen), *n* = 324	High-grade Nonhigh-grade	93 (61.6) 126 (72.8)	58 (38.4) 47 (27.2)	1.672 (1.046, 2.672)	0.033

Suspect (micro)invasion in the biopsy specimen, *n* = 349	Present Absent or not mentioned	19 (36.5) 212 (71.4)	33 (63.5) 85 (28.6)	4.332 (2.335, 8.036)	<0.001

**Table 2:** Specific score numbers for variables used in the predictive model; the total score in an individual case is the sum of the corresponding specific score numbers.

Category of the variable	Specific score number
Suspected (micro)invasion in the biopsy specimen	13.3
The lesion is visible on ultrasonography	10.6
The size of the lesion on mammography >30 mm	9.2
The lesion is palpable	9.2

**Table 3:** The probability of having invasive cancer in the resection specimen (probability of invasion) as a function of total score.

Total score	Probability of invasion
0	0.09
9.8	0.20
12.6	0.25
15.2	0.30
23.7	0.50
34.6	0.75

**Table 4:** Assessment of the performance of the predictive model in the test population, *n* = 204.

Association of predicted probabilities and observed responses			
Percent concordant	69.2	Somers' D	0.511
Percent discordant	18.0	Goodman-Kruskal Gamma	0.586
Percent tied	12.8	Kendall's Tau-a	0.230
Pairs	9315	Area under ROC	0.756

**Table 5:** Performance of the simplified model in the test population, *n* = 204.

	Number of patients with invasion detected in the resection specimen (%)	Number of patients with pure DCIS in the resection specimen (%)	Positive predictive value (95% CI)	Negative predictive value (95% CI)
Low probability predicted (low-risk group), *n* = 107	18 (16.8)	89 (83.2)	52.6% (42.6, 62.5)	83.2% (76.1, 90.3)
High probability predicted (high-risk group), *n* = 97	51 (52.6)	46 (47.4)		

**Table 6:** Assessment of the performance of the predictive model in the validation population, *n* = 71.

Association of predicted probabilities and observed responses			
Percent concordant	80.6	Somers' D	0.705
Percent discordant	10.1	Goodman-Kruskal Gamma	0.777
Percent tied	9.3	Kendall's Tau-a	0.260
Pairs	918	Area under ROC	0.852

**Table 7:** Performance of the simplified model in the validation population, *n* = 71.

	Number of patients with invasion detected in the resection specimen (%)	Number of patients with pure DCIS in the resection specimen (%)	Positive predictive value (95% CI)	Negative predictive value (95% CI)
Low probability predicted (low-risk group), *n* = 42	3 (7.1)	39 (92.9)	48.3% (30.1, 66.5)	92.9% (85.1, 100.0)
High probability predicted (high-risk group), *n* = 29	14 (48.3)	15 (51.7)		
